# Supplementary material for: Impaired Function of PLEKHG2, a Rho-Guanine Nucleotide-Exchange Factor, Disrupts Corticogenesis in Neurodevelopmental Phenotypes
Source: Cells. 2022 Feb 16;11(4):696. doi: 10.3390/cells11040696 (PMC8870177; doi:10.3390/cells11040696)
Supplement: Supplementary file 1 [file cells-11-00696-s001.zip › cells-1580958-Supplementary Figures.pdf]

Supplementary information

Impaired function of PLEKHG2, a Rho-guanine nucleotide-exchange factor, disrupts corticogenesis in neurodevelopmental phenotypes

Masashi Nishikawa, Hidenori Ito, Hidenori Tabata, Hiroshi Ueda and Koh-ichi Nagata

Materials and methods

1. Antibodies and reagents

The following antibodies were used: anti-GFP (Medical & Biological Laboratories, Cat# 598, RRID: AB_591819 or Nacalai Tesque, Cat# 04404-84, RRID: AB_10013361) and anti-5-bromo-2’-deoxyuridine (BrdU) (Roche, Cat# 10875400). Alexa Fluor 488 and 647 (Invitrogen) were used as secondary antibodies. 4’, 6-diamidino-2-phenylindole (DAPI; Nichirei Bioscience, Tokyo, Japan) was used for staining DNA. 5-ethynil-2’-deoxyuridine (EdU) were detected with Alexa Fluor 555 azide (Life Technologies).

2. Cell proliferation experiments

Analyses were done as previously reported [1]. E14 embryos were electroporated with pCAG-EGFP (0.5 μg) together with sh-Luc or sh-Plek2#2 (1.0 μg each), and their mothers were injected with BrdU (Wako; 50 μg/g of body weight) 30 h later. Three hours after BrdU injection, EdU (Life Technologies; 25 μg/g of body weight) was also administered into the mother, which was killed 1 h later. Brains were then fixed with 4% paraformaldehyde and 14 μm thick sections were cut with a Cryostat. For BrdU immunolabeling, sections were first incubated with 2N HCl at 37 ℃ for 30 min to unmask the antigen, followed by three washes in PBS. Sections were blocked with 10% BSA in PBS and 0.5% Triton X-100 for 1 h, and then stained in antibody (see above). Sections were photographed with an LSM-880 confocal laser microscope (Carl Zeiss), and acquired images were analyzed with ImageJ to count cells in a blinded manner. Statistical analyses were performed using R.

Supplementary data

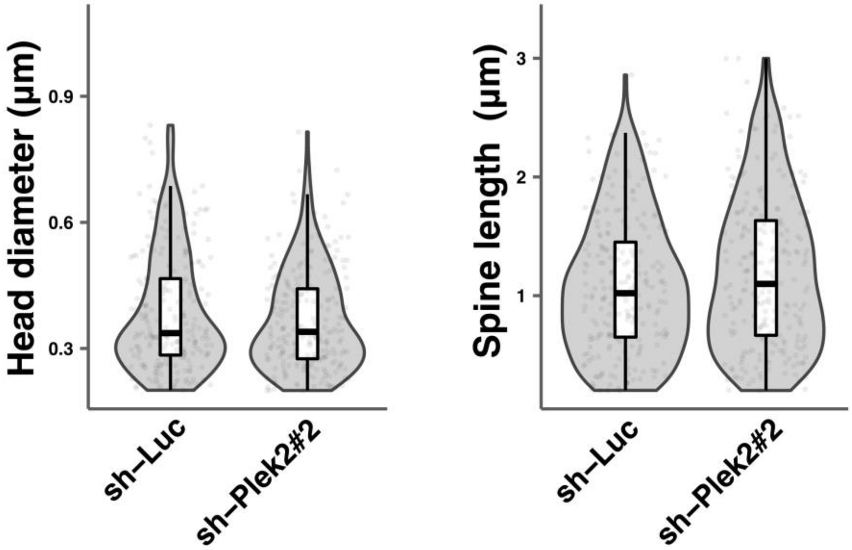

Supplementary figure S1. Role of Plekhg2 in the dendritic spine morphology *in vivo*.

Note that knockdown of Plekhg2 little affected the spine length and head diameter.

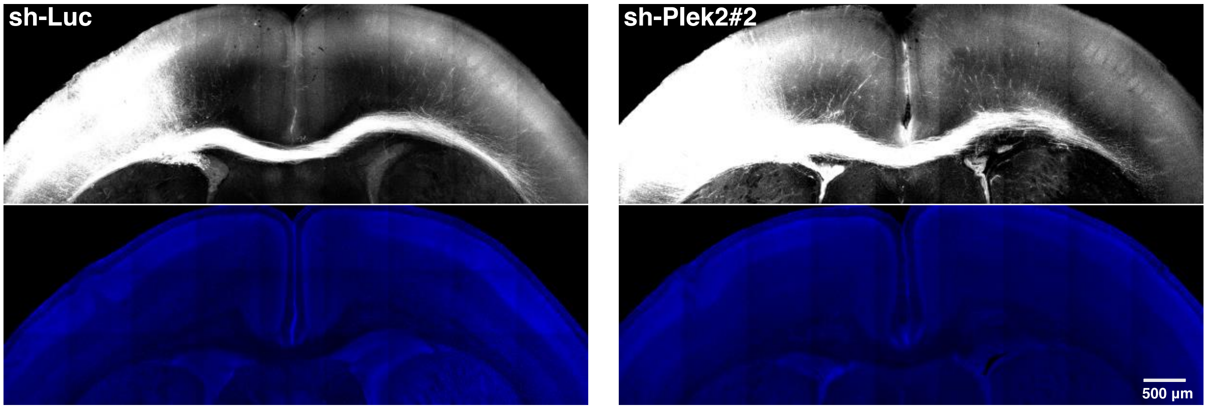

Supplementary figure S2. Role of Plekhg2 in the axon extension *in vivo.*

pCAG-EGFP (0.5 µg) was co-electroporated *in utero* with sh-Luc or sh-Plek2#2 (1.0 µg each) into the VZ progenitor cells at E14.5. Coronal sections were prepared at P7, and were double-stained with anti-GFP (*white*) and DAPI (*blue*). Note that, as in the case of control neurons, Plekhg2-deficient neurons extended axon bundles to the contralateral cortex.

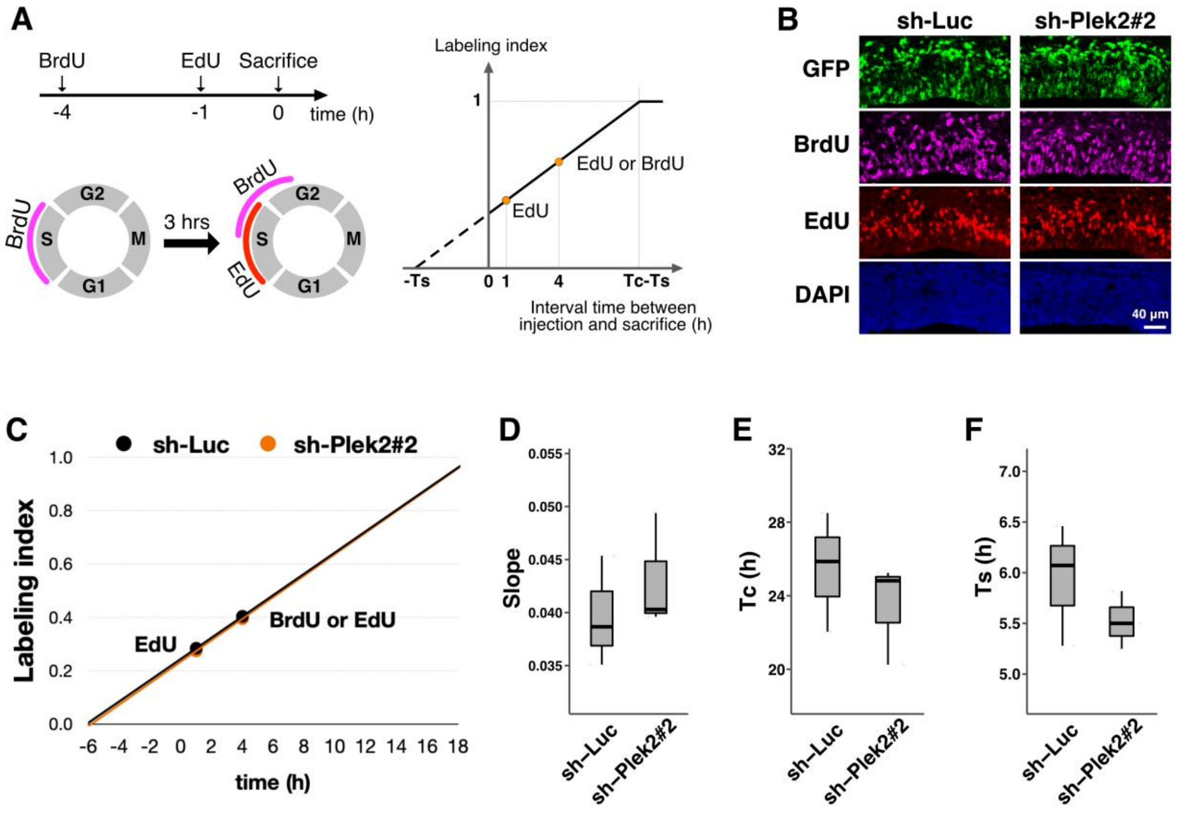

Supplementary figure S3. Estimation of the length of the cell cycle of Plekhg2-deficient cortical progenitors.

Analyses were done as previously reported [1]. **(A)** BrdU was administrated 4 h before the mice were sacrificed at E15.5 and EdU 3 h later. The availability of BrdU and EdU for incorporation in dividing cells is estimated at 2 h. The time it takes to label all cells is Tc - Ts, which is the time it takes for a cell that was just at the end of the S-phase at the time of the first injection to pass through G2, M, and G1 and then reenter S-phase. Tc and Ts are estimated based on the proportion of GFP-positive cells that were proliferating 1 or 4 h before the mice were sacrificed. **(B)** Coronal sections were stained with anti-GFP (*green*), anti-BrdU (*magenta*), anti-EdU (*red*), and DAPI (*blue*). **(C - F)** Counting the number of GFP-positive cells in the VZ that are EdU-positive or BrdU/EdU-double positive showed that Plekhg2-knockdown had little effects on neuronal precursor proliferation.

Reference

1. Friocourt, G.; Kanatani, S.; Tabata, H.; Yozu, M.; Takahashi, T.; Antypa, M.; Raguénès, O.; Chelly, J.; Férec, C.; Nakajima, K.; et al. Cell-autonomous roles of ARX in cell proliferation and neuronal migration during corticogenesis. *J. Neurosci.* **2008**, *28*, 5794–5805, doi:10.1523/JNEUROSCI.1067-08.2008.
